# Unlocking the potential of targeting the angiotensin II type 1 receptor in cancer

**DOI:** 10.1038/s41388-025-03666-9

**Published:** 2025-12-17

**Authors:** David R. Butcher, Christopher N. Parris, Scott J. Crichton, Fiona C. Dempsey, Hussein N. Al-Ali

**Affiliations:** 1 Anglia Ruskin University, Cambridge, UK; 2 aTen Therapeutics Ltd, Edinburgh, Scotland UK

**Keywords:** Drug development, Cancer microenvironment

## Abstract

The renin-angiotensin system is a key regulator of blood pressure homeostasis, with its primary effector, the angiotensin II type 1 receptor (AT1R), mediating vasoconstriction and processes fundamental to cancer progression, including proliferation, angiogenesis, and metastasis. Elevated AT1R expression is consistently linked to poor prognosis and therapeutic resistance across various malignancies. Preclinical studies provide compelling evidence that AT1R activation drives key cancer related processes, while its inhibition by angiotensin receptor blockers (ARBs) suppresses tumour growth, induces apoptosis, reduces angiogenesis, and inhibits metastasis across a wide range of cancer models. Critically, ARBs effectively modulate the tumour microenvironment (TME), alleviating fibrosis, promoting anti-tumour immune cell phenotypes, and enhancing the efficacy of targeted therapies, chemotherapies, and immunotherapies. Despite this strong preclinical evidence and supporting retrospective population studies, clinical translation of ARBs in oncology remains inconsistent, with trials often limited by design, patient heterogeneity, and supra-therapeutic ARB dosages required for acute anti-cancer effects. This review seeks to summarise the current understanding of AT1R’s role in cancer, highlight preclinical and clinical investigations of targeting RAS, and suggest further strategies to unlock its therapeutic potential. Realising the full therapeutic promise of AT1R targeting in oncology requires a multifaceted approach, including the development of innovative delivery systems, such as TME-activated ARBs, and the exploration of advanced therapeutic modalities, such as antibody based AT1R inhibitors. Rigorously designed clinical trials that include biomarker-driven patient stratification to identify responsive cohorts are crucial to define the context-dependent role of AT1R and conclusively establish its clinical utility as a combinatorial strategy to enhance patient outcomes.

## The renin-angiotensin system

The renin-angiotensin system (RAS) is an endocrine system that is a key regulator of blood pressure through the modulation of fluid volume homeostasis, electrolyte balance, and vascular structure/integrity. Once thought to signal only at the systematic level, the RAS is now understood to be regulated both locally, through cell and tissue interaction, and systematically via endocrine mechanisms. These two modes of regulation are referred to as the local and systemic RAS, respectively [1, 2].

### Angiotensin structure, metabolite formation, and receptor function

**Fig. 1:**
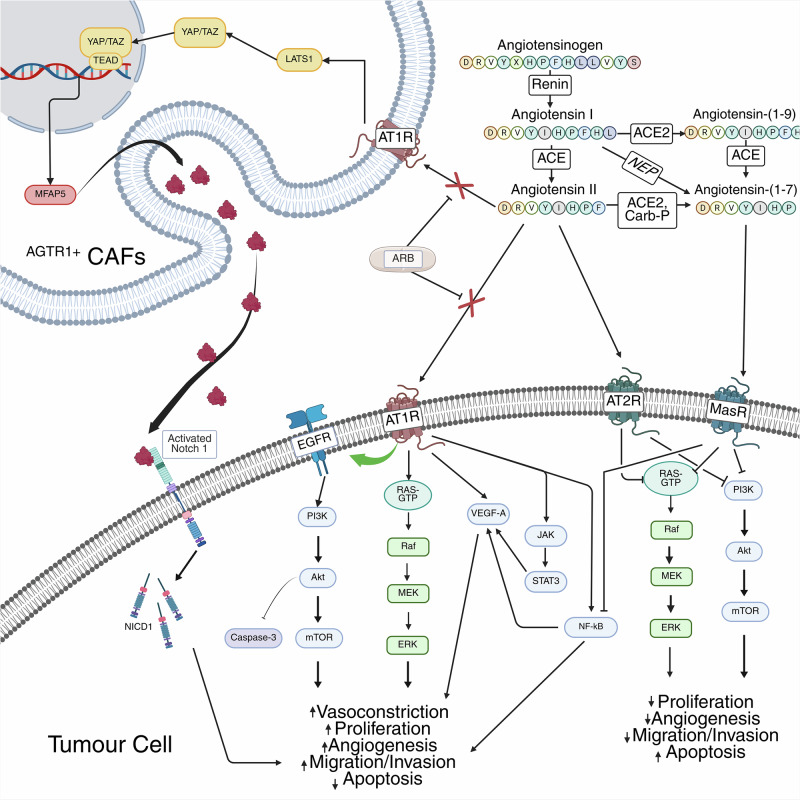
Renin-angiotensin system signalling in cancer. Angiotensinogen is enzymatically cleaved into bioactive peptides which activate GPCRs, AT1R, AT2R and MasR. AT1R signalling induces various cancer related processes in both tumour cells and CAFs via various intracellular signalling cascades and EGFR transactivation. The counter-regulatory arm of the RAS, AT2R and MasR, inhibits various intracellular signalling cascades to reduce cancer related processes. Angiotensin receptor blockers specifically inhibit AT1R signalling, reducing pro-cancer signalling and simultaneously increasing anti-cancer signalling by freeing Ang II to bind to AT2R or be converted into Ang 1-7.

The primary precursor of the RAS, angiotensinogen (Agt) [2, 3], is a 57 kDa glycoprotein mainly produced in the liver. Agt is the only known substrate for the aspartyl protease, renin, which is secreted from the juxtaglomerular cells of the kidneys in response to reduced plasma sodium or fluid volume levels. It is sequentially cleaved by renin into the inactive decapeptide angiotensin I which is then further cleaved by angiotensin-converting enzyme 1 (ACE1), producing the bioactive octapeptide angiotensin II (Ang II (Ang1–8)) (Asp-Arg-Val-Tyr-Ile-His-Pro-Phe) [1, 2]. Further enzymatic cleavage generates additional bioactive peptides, which exert their effect via binding to various G-protein-coupled receptors (GPCR). The principal effector of the RAS is the 359 amino-acid, 41 kDa, seven-transmembrane GPCR, angiotensin II type 1 receptor (AT1R), encoded by the *AGTR1* gene [1]. This complicated regulatory system involving these peptide-receptor interactions and their downstream signalling (Fig. 1) has previously been thoroughly reviewed [4].

The classical RAS axis involving ACE1/Ang II/AT1R mediates vasoconstriction, as well as many functions associated with cancer progression, including proliferation and angiogenesis. Due to this vasoconstrictive function, many small molecule inhibitors of ACE1 (-prils) and AT1R (-sartans) have been developed to treat hypertension (Table 1). The non-classical pathways (including Ang II/AT2R, Ang [1–7]/MasR and alamadine/MRGD) regulate many of the same processes as the classical pathway, often exerting opposing effects, reducing proliferation, and promoting vasodilation [1]. Further complicating this intricate system, GPCRs operate not only as monomers but often function in dimeric (homo and hetero) and even oligomeric states. Similarly, RAS receptors have been shown to form heteromers with many other receptors, which mediate many of the functions of both classical and non-classical RAS signalling [4]. For example, AT1R can transactivate receptor tyrosine kinases, including epidermal growth factor receptor (EGFR). Activation of AT1R induces EGFR signalling, modulating the intensity and duration of EGFR downstream signalling. This activation is inhibited by the ARB losartan or inhibition of the direct interaction between AT1R and EGFR [5, 6].

**Table 1:** List of clinically approved renin angiotensin system inhibitors.

Target	ACE			AT1R			Renin
**Drug name**	captopril	imidapril	zofenopril	candesartan	telmisartan	valsartan	aliskiren
	cilazapril	moexipril	enalapril	eprosartan	olmesartan	losartan	
	perindopril	quinapril	ramipril	irbesartan	azilsartan		
	lisinopril	spirapril	benazepril				
	delapril	fosinopril	trandolapril				

Given the role of AT1R in fundamental physiological processes and interactions with known cancer-related signalling pathways, it is unsurprising that AT1R dysregulation has been implicated in cancer development, progression, and response to anti-cancer therapy [7]. Downstream activation of effectors, phospholipase A, C and D, mitogen-activated protein kinase, protein kinase B (PKB/Akt) and protein kinase C (PKC), as well as epidermal growth factor (EGF) receptor (EGFR) transactivation make Ang II/AT1R signalling a potent mitogenic signal, resulting in proliferation, cell migration and angiogenesis [1, 7]. When dysregulated, these processes are all hallmarks of cancer [8], identifying AT1R signalling as a strong candidate for cancer therapy.

## The RAS in cancer

Cancer is a leading cause of mortality worldwide, accounting for 9.7 million deaths in 2022, with 1 in 9 men and 1 in 12 women dying from cancer. There were also an estimated 20 million new cancer cases in the same period, indicating a huge global burden [9]. Whilst advances in our understanding and treatment of these diseases have reduced mortality rates, enhanced therapeutic strategies remain a necessity to reduce the socio-economic burden of cancer. Unlocking the potential of targeting AT1R as a cancer treatment may provide a new strategy in the arsenal of anti-cancer therapeutics.

### Population studies and initial identification of AT1R/RAS as a cancer target

Elevated expression of AGTR1, the gene encoding AT1R, has been linked to poor-prognosis [8] and chemotherapy resistance in breast cancer [10], and lower progression free-survival outcomes in glioblastoma [11], colorectal cancer (CRC) [12], hepatocellular carcinoma (HCC) [13], and oesophageal squamous cell carcinoma (OSCC) [14]. Population studies investigating the association of RAS inhibitor (RASi) use, ARBs, and ACE inhibitors (ACEis), with cancer incidence or survival have yielded mixed, yet mostly positive results (Table 2) [10, 12, 13, 15–41]. Whilst some studies found an increased risk with RASi use, particularly with ACEis or grouped RASis, many studies provided evidence of a potential benefit of ARB use [42], reducing risk, improving outcomes and increasing survival in a variety of cancers, including colorectal [24], kidney [25], liver [26, 27], lung [19–23, 33] and breast [41]. These contradictory results suggest that indiscriminate inhibition of RAS signalling prevents some beneficial signalling pathways, such as Ang 1–7/MasR, whereas targeted AT1R inhibition allows these beneficial signalling pathways to remain active. This highlights the need for further investigation into the specific effects of different RASis, and their potential role in cancer treatment, with ARBs and ACEis separated into distinct groups.

**Table 2:** Population study data of renin-angiotensin system inhibitor (RASi) use and cancer risk/survival in various cancer subtypes.

Cancer type	Therapeutic assessed	Hazard ratio (HR), Odds ratio (OR), or Relative Risk (RR)	Type of study	Ref.
Overall cancer	ARB and ACEi	ARB HR = 0.83, 95% CI [0.74, 0.93] ACEi HR = 0.92 95% CI [0.86, 0.99]	Meta-analysis	[42]
-	ACEi	OR = 1.269 95% CI [1.088, 1.480]	Retrospective cohort study	[36]
Breast	RASi	RR = 0.99, 95% CI [0.93, 1.05] RR ( > 10 years RASi use) = 0.80, 95% CI [0.67, 0.95]	Meta-analysis	[41]
Colorectal CRC	RASi	RR = 0.86, 95% CI [0.78, 0.93]	Meta-analysis	[24]
CRC	RASi	HR ( < 3 years post index colonoscopy) = 0.78, 95% CI [0.64, 0.96]	Retrospective cohort study	[28]
Kidney	ARB	HR = 0.818, 95% CI [0.691, 0.969]	Meta-analysis	[31]
mRCC	RASi	HR (OS) = 0.81, 95% CI [0.707, 0.929]	Pooled analysis	[25]
mRCC (Sunitinib treated)	RASi	HR (OS) = 0.40, 95% CI [0.24, 0.66]	Retrospective cohort study	[30]
Liver HCC	RASi	HR = 0.6, 95CI [0.4, 0.9]	Retrospective cohort study	[26]
HCC	RASi	HR (OS) = 0.50, 95% CI [0.34, 0.74]	Retrospective cohort study	[27]
Lung	ARB	RR = 0.81, 95% CI [0.69, 0.94]	Meta-analysis	[19]
mNSCLC	RASi	HR = 0.72, 95% CI [0.55, 0.95]	Retrospective cohort study	[23]
Oral OSCC	ARB	HR (OS, Advanced OSCC) = 0.61, 95% CI [0.39, 0.94]	Retrospective cohort study	[29]
Pancreatic	ARB	HR (OS) = 0.80, 95% CI [0.72, 0.89]	Retrospective cohort study	[32]
Gastric Gastro-oesophageal	ARB	HR = 0.83, 95% CI 0.71, 0.98] HR ( > 2 years ARB use) = 0.42, 95% CI [0.25, 0.72]	Retrospective cohort study	[34]
Prostate	ACEi	OR = 1.438, 95% CI [1.090, 1.897]	Retrospective cohort study	[36]

*ACEi* Angiotensin converting enzyme inhibitor, *ARB* Angiotensin receptor blocker, *CI* Confidence interval, *CRC* Colorectal carcinoma, *HCC* Hepatocellular carcinoma, *HR* Hazard ratio, *mNSCLC* Metastatic non-small cell lung cancer, *mRCC* Metastatic renal cell carcinoma, *OS* Overall survival, *OSCC* Oral squamous cell carcinoma, *OR* Overall risk, *RASi* Renin-angiotensin system inhibitor, *RR* Relative risk.

Many studies show a positive impact of RASi use on cancer risk and overall survival. The studies that show an increased risk/mortality implicate either RASi or specifically ACEi.

### Preclinical evaluation of RASi in cancer

Investigations into the role of AT1R signalling and the effect of various ARBs in an array of pre-clinical models have been undertaken and generated compelling data to support the therapeutic potential of targeting AT1R in cancer. These studies have concluded that AT1R signalling is implicated in promoting many hallmarks of cancer [8], with inhibition by ARBs found to inhibit these processes (Fig. 1). Inhibition of AT1R can also increase the efficacy of other chemotherapeutics and overcome therapy resistance [43]. Furthermore, some studies have found upregulation of AT2R [44] and Ang 1–7 [45] provide an anti-cancer effect, indicating a role for other RAS components and supporting the hypothesis that indiscriminate inhibition of the RAS is not viable as a therapeutic strategy in cancer.

#### Cancer cell proliferation

AT1R activation is often described as a potent mitogenic signal, triggering downstream effectors and intracellular signals linked to increased cancer cell proliferation and tumour growth in a wide variety of cancer types [16, 46–48]. Pharmacological or genetic inhibition of AT1R has been shown to reduce proliferation and increase apoptosis via a wide variety of downstream signalling pathways (Fig. 1).

The PI3K/AKT/mTOR pathway is often implicated in enhancing mitogenic signalling. EGFR signalling is known to stimulate this pathway and it is likely that AT1R mediated EGFR-transactivation promotes the proliferative effects of AT1R in cancer [5, 6]. Targeted AT1R inhibition-mediated downregulation of the PI3K/AKT/mTOR pathway has a role in reduced proliferation and increased apoptosis in CRC [46], lung [47], kidney [48], ovarian [49], and oesophageal [16] cancers, as demonstrated by reduced cell viability and tumour volume [46], reduced Ki-67 and increased TUNEL staining [47, 48]. The transcription factor NF-κB, downstream of PI3K/AKT/mTOR pathway, is suppressed by ARBs such as losartan and azilsartan in breast cancer, correlating with reduced proliferation and increased apoptosis [8, 47]. In vivo, AGTR1 upregulation in breast cancer increased metastasis, while ARBs reversed these effects [50]. These findings were also observed in HCC and lymphoma mouse models [51, 52].

AT1R inhibition also suppresses MAPK and ERK signalling. Both ARBs and gene silencing of AT1R reduce p-ERK levels and, consequently, proliferation in gastric [53], ovarian [49] and pancreatic [54] cancer cells, with these effects replicated in xenograft models [49, 54]. Similar effects were observed with AT2R overexpression in bladder cancer, suggesting ARB treatment biases Ang II to AT2R and results in tumour suppression [44]. Telmisartan inhibits JNK, a subfamily of MAPK, inhibiting downstream c-Jun expression in a HIPO/YAP1-dependent manner [43]. HIPPO/YAP1 signalling is also implicated in Ang II-mediated proliferation in intrahepatic cholangiocarcinoma (iCCA), with ARBs disrupting AGTR1+ cancer-associated fibroblast (CAF) MFAP5/Notch1 signalling by impeding YAP/TEAD nuclear translocation and reducing tumour proliferation (Fig. 1) [55].

Cell cycle arrest is another mechanism downstream of AT1R inhibition. ARBs downregulate cyclin D1 across multiple cancer models [56–59], and telmisartan reduces cyclin A2 and CDK2 in oesophageal cancer xenografts [60]. AT1R inhibition has been shown to induce G0/G1 [53], G2/M [48], and S-phase [60] arrests, although the precise mechanism and cell type specificity is not yet fully understood. Despite this, cell cycle inhibition is a recurring hallmark of the anti-tumour effects of AT1R inhibition.

#### Metastasis/Migration/invasion

AT1R activation drives cellular processes critical for cancer progression and metastasis, including migration, invasion, and epithelial-mesenchymal transition (EMT), across multiple tumour types. In breast cancer, AGTR1-overexpressing MCF-7 cells exhibited elevated EMT markers (p-Smad, Smad4, Snail) as well as enhanced migration and invasion. In vivo, these cells formed xenografts with reduced E-cadherin and increased vimentin and matrix-metalloprotease 9 (MMP-9) expression [61]. Conversely, ARBs reduced colony formation, migration, and lung metastases in xenograft models of breast cancer [61, 62]. In contrast, Ang II was found to only exert an impact on fibroblasts co-cultured with 4T1-Luc cells and not 4T1-Luc cells alone [59], implicating stromal involvement in tumour EMT. Furthermore, prophylactic losartan treatment was shown to reduce ductal carcinoma in situ (DCIS) progression and correlated with lower IL-6 and p-STAT3 expression [15]. Losartan also inhibited lymph node metastases via downregulation of CXCR4/SDF-1α and downstream FAK/RhoA signalling [50].

In CRC, Ang II increased ZEB1 expression and promoted migration. Whilst treatment with both irbesartan and an AT2R inhibitor inhibited migration, only irbesartan reversed Ang II-induced ZEB1 and vimentin expression and E-cadherin downregulation. These results indicate an AT1R-specific effect on EMT drivers such as ZEB1, vimentin, and E-cadherin [63]. Similarly, losartan and candesartan inhibited CRC cell migration, inhibiting MMP-3 and MMP-9 expression while restoring E-cadherin [46, 57]. These effects were validated In vivo, where irbesartan reduced ZEB1-positive infiltrating cells in CRC liver metastases, and valsartan decreased lung metastases in CT-26 xenografts [63, 64].

Similar AT1R-mediated effects on EMT, migration and invasion have been reported in a wide variety of cancer subtypes [65]. Telmisartan reduced IL-6 expression in gastric cancer [66], and in lung cancer lowered TGFβ while increasing E-cadherin expression [67]. Ang II upregulated MMP-2, -9, and -14 in lymphoma, which was reversed by valsartan treatment [68]. Additionally, in prostate cancer, AT1R-agonistic autoantibodies have been shown to enhance invasion [40]. While AT2R and MasR have been implicated in EMT in CRC and ovarian cancers [63, 69], AT1R appears to have a more significant role in ovarian cancer, where its overexpression significantly increased migration [49].

Collectively, these findings support a model in which AT1R promotes EMT, invasion, migration, and metastasis through regulation of transcription factors, extracellular remodelling, and immune/stromal modulations. The interplay between AT1R, AT2R and MasR may shape these effects, but AT1R blockade remains central to limiting metastatic progression across cancer types.

#### Angiogenesis

Angiogenesis, the formation of new blood vessels from pre-existing vasculature, is critical for tumour growth, invasion, and metastasis [70]. While AT1R-dependent angiogenesis is well defined in cardiovascular tissue [1], increasing evidence implicates AT1R in pathological angiogenesis across several cancer types.

In breast cancer, AT1R expression correlates with higher vascular density [17]. Analysis of The Cancer Genome Atlas (TCGA) data further linked AGTR1 to angiogenesis-related NF-κB gene targets [71], supported by in vitro and In vivo studies showing increased vascularisation via CARMA3/Bcl10/MALT1 signalling and inhibition of p-IκB by losartan [10]. Similarly, AGTR1 overexpression increased angiogenesis and microvessel density (MVD), while losartan reversed these effects. It has also been noted that prophylactic losartan reduced progression from DCIS to invasive cancer, increasing vessel diameter but not number, whilst reducing VEGFa levels [15].

Telimisartan has been shown to downregulate Bcl-2, previously associated with VEGF induction, in lung cancer cells [47, 72], whereas losartan was found to inhibit Ang II-induced VEGFA and IL-8 expression in liver cancer [73]. Additionally, HCC tissue showed high AT1R levels, correlating with VEGFa and MVD which candesartan treatment was able to reduce [74]. It was also demonstrated that Ang 1–7 inhibits VEGF expression and MVD in both lung and liver cancer [75]. In both these studies, Ang 1–7 treatment downregulated AT1R expression, with a ~ 4-fold reduction of AT1R mRNA observed in liver cancer [45]. The effect of Ang 1–7 in lung cancer was only partially supressed by a MasR inhibitor, implicating AT1R downregulation in the total effect [75].

Other cancer subtypes show similar trends. Losartan reduced VEGF and CD34 expression in mRCC [76], though lower-dose treatment paradoxically increased vascular permeability [77]. miR-410 (micro-RNA targeting AT1R mRNA) reduced CD31 staining in pancreatic cancer xenografts [54], further implicating AT1R in angiogenesis. Whilst the precise mechanisms remain to be fully understood, these studies collectively support a broad role for AT1R in tumour vascularisation across multiple cancer types.

#### Tumour microenvironment effects

Angiogenic and metastatic processes contribute to the conditions of the tumour microenvironment (TME), a complex, active driver of cancer progression, composed of immune and stromal cells, extracellular matrix, and blood vessels [78]. Given its role in processes such as EMT [61], inflammation [15], fibrosis [46] and angiogenesis [61], AT1R signalling directly contributes to TME maturation.

Losartan has been shown to reduce the mRNA expression of several TME-related proteins, including TGFβ1, integrin β3, CTGF, IL-1, IL-4, IL-10, TNFα, and MIP-1α/CCL3 [15]. In tumour-bearing mice, ACEi treatment suppressed elevated serum levels of TGFβ1, Il-2, Il-4, Il-10, and TNFα [79]. Interestingly, tumour suppression was dependent on neutrophils, as this effect was eliminated in neutrophil-depleted mice. Administration of captopril was shown to promote anti-tumour neutrophil phenotypes that reduced tumour growth in untreated mice after adoptive transfer of splenic cells from treated animals [79]. This supports other studies where Ang II was found to have no effect on 4T1-Luc cells in vitro, but co-culture with fibroblasts revealed an Ang II-driven increase in fibronectin, vimentin, and α-SMA [59] - a marker of dense stroma that impairs T-lymphocyte recruitment and is downregulated by losartan [80]. Losartan has also been shown to promote macrophage polarisation from pro-tumoural M2-like to anti-tumoural M1-like phenotypes [81], as well as alleviate stromal density by reducing collagen, α-SMA, TGFβ, and HIF-1α [81, 82].

Further modulation of immunosuppressive cells by ARBs has also been observed. ARBs counteract the effects of CAFs, likely via inhibition of TGFβ and IL-10 [82]. These CAFs impaired T-cell recruitment, while ARB treatment upregulated several T-cell activation markers [82]. Metastasis-associated fibroblast (MAFs) in colorectal cancers have been shown to express elevated levels of Agt and AT1R [83] with Ang II enhancing MAF-mediated extracellular matrix (ECM) remodelling. In contrast, dual inhibition with captopril and losartan inhibited this effect [83]. The lack of significant effect of AT2R activation or inhibition on ECM remodelling suggests that this process is primarily driven by an ACE/Ang I/AT1R-mediated mechanism [83]. In intrahepatic cholangiocarcinoma (iHCC), losartan reduced stromal density by inhibiting YAP1/LAT1 dephosphorylation and MFAP5-mediated Notch1 signalling from AT1R+ CAFs [55] (Fig. 1). Data obtained also demonstrated that losartan depleted immunosuppressive CAFs and increased CD8 + T-cell infiltration [84].

Consistent with these findings, losartan and candesartan reduced fibrosis in CT-26 xenograft models of CRC [46, 57, 64]. Although one study reported increased TNFα expression after losartan treatment [46], this contradicts broader findings, suggesting methodological variability [57, 85]. Candesartan also outperformed 5-FU in reducing collagen deposition [57]. Losartan additionally inhibits the recruitment of metastasis-promoting, inflammatory monocytes. Intriguingly, this was mediated via CCR2, as evidenced by a similar effect in AGTR⁻/⁻ mice, raising the possibility of losartan-mediated off-target effects [86]. It is worth noting, however, that mice have 2 isoforms of AT1R that are products of separate genes (AGTR1a and AGTR1b), and that the AGTR⁻/⁻ mice used in this study were only shown to be negative for AGTR1a, and not AGTR1b [87], indicating a possible effect on this, yet to be fully understood, ortholog of AT1R.

Together, these studies provide compelling evidence for the role of ARBs, if not AT1R signalling directly, in shaping TME architecture and function. Targeting this pathway should help alleviate a tumour suppressive TME, particularly through modulation of fibroblast activity, immune cell recruitment, and stromal remodelling.

#### Combination with other anti-cancer therapeutics

Therapeutic resistance remains a significant barrier to effective cancer treatment, often requiring combination therapy to overcome the adaptive escape mechanisms that cancer cells initiate [88]. Due to their role in modulating the TME, angiogenesis, inflammation and proliferation, ARBs are emerging as mechanistically supportive adjuncts across therapeutic classes.

##### Targeted therapy

Despite tyrosine kinase inhibitors (TKIs), such as everolimus or sunitinib in RCC [89], forming a critical class of targeted therapies that modulate key oncogenic signalling cascades, their use is limited due to the development of resistance and adverse effects [90]. RAS inhibition, particularly via ARBs, may enhance TKI tolerability and efficacy. Retrospective studies have shown that RASi use correlated with improved OS and PFS in mRCC patients receiving sunitinib, an anti-VEGFRs and PDGFRs therapeutic (Table 2), likely through mechanisms beyond blood pressure control [30]. In HCC, RASi combined with sorafenib, an anti-VEGF TKI, improved median OS compared to monotherapies (19.5 vs 10.9 vs 9.7 months, RASi + sorafenib vs RASi vs sorafenib) [26]. Meanwhile, use of RASi and EGFR-TKI combinations in NSCLC patients suggested a trend toward longer PFS, though OS differences were not significant, possibly due to small samples sizes [91, 92].

These results are corroborated in vitro and in vivo, where losartan combined with lenvatinib (anti-VEGFR TKI) reduced endothelial and tumour cell proliferation, angiogenesis, and tumour burden in Huh7 xenografts [73]. Conversely, although losartan mitigated axitinib (VEGFR inhibitor)-induced hypertension, it did not enhance tumour suppression, indicating mechanistic overlap [77]. Other studies have shown telmisartan reduced stem cell markers in rociletinib-resistant H1975 cells, with the combination of telmisartan, CFM 4.16, and sorafenib significantly reducing H1975-xenograft tumour volume [67]. Mechanistically, telmisartan suppressed EGFR and MET phosphorylation, implicating AT1R inhibition in resistance modulation [67].

##### Chemotherapy

Cytotoxic chemotherapies, including DNA-damaging agents (e.g., 5-FU, cisplatin, and doxorubicin) and mitotic inhibitors (e.g., paclitaxel), remain foundational therapies in cancer care but are limited by toxicity and resistance [93].

Doxorubicin, though effective, induces cardiotoxicity [94]. Valsartan reduced doxorubicin-induced reactive oxygen species (ROS) and apoptosis in cardiomyocytes without impairing its anti-cancer activity. Furthermore, co-culture with mesenchymal stem cells enhanced this protective effect [94]. ARBs also reduced doxorubicin-induced cardiotoxicity In vivo, potentially via TGFβ and MAPK modulation [95, 96]. In addition, acute myeloid leukaemia cells with immune/apoptotic gene signatures have been shown to become more sensitive to doxorubicin after ARB treatment [97].

5-fluorouracil (5-FU), an inhibitor of DNA synthesis, has shown synergy with valsartan in CRC models, increasing apoptosis, (increased levels of Bax and p53 plus decreased Bcl2 levels found), inhibiting migration (decreased MMP-2 and MMP-9 levels) and reducing tumour burden In vivo, though not as significantly as 5-FU monotherapy. Combination therapy reduced VEGF, Col1A1, and IL-6 expression and reduced fibrosis [64]. Both losartan and candesartan demonstrated synergy with 5-FU in fibrosis suppression, though TNFα expression was variably modulated depending on which ARB was utilised, indicating a drug-specific mechanism [46, 57]. ARBs also reduced 5-FU side effects with losartan reducing mucositis [85] whilst telmisartan mitigated cachexia and simultaneously improved tumour response in gastric cancer [66]. Losartan also synergistically enhanced 5-FU-induced growth inhibition in OSCC [98].

Gemcitabine and nab-paclitaxel (GEM/AB) are a standard of care therapy in pancreatic ductal adenocarcinoma (PDAC). Irbesartan resensitised PDAC cells and organoids, improved GEM/AB efficacy, and reduced tumour growth in a variety of models, from in vitro cell lines to patient-derived organoid xenografts [43]. Irbesartan reduced nuclear YAP1, supressed c-Jun and decreased stemness and iron metabolism [43]. Other ARBs also enhanced paclitaxel activity, particularly at low doses [99].

In NSCLC patients, RASi improved survival in carboplatin/paclitaxel-treated patients, but not when bevacizumab (anti-VEGF) was included, suggesting overlapping anti-angiogenic effects of bevacizumab and RASi [100]. This supports observation that platinum-resistant NSCLC cells overexpress AT1R and VEGF, with olmesartan treatment suppressing growth in platinum-resistant tumours [101]. Additionally, Ang 1–7, possibly acting through AT1R downregulation, inhibited growth in platinum-resistant xenografts [75]. Paclitaxel efficacy was also enhanced by losartan in ovarian cancer models, likely by reducing ECM stiffness and improving drug penetration [80].

Due to the nature of chemotherapeutics being used in combination, it is difficult to untangle the precise mechanism of the increased efficacy seen with concomitant ARB treatment. Despite this, ARBs have been shown to directly augment the effect of DNA-damaging agents (doxorubicin, gemcitabine, and 5-FU) individually as well as the anti-mitotic, paclitaxel. These results are corroborated in combined therapies, indicating that targeting AT1R signalling is a useful addition to the arsenal of chemotherapeutic regimes, boosting efficacy and reducing side effects, potentially resulting in improved survival and quality of life.

##### Immunotherapy

Immune checkpoint inhibitors (ICIs) have revolutionised cancer therapy, yet TME-related barriers, such as dense stoma and fibrosis, limit their efficacy. AT1R inhibition may relieve these constraints. Unsurprisingly, retrospective cohort studies found that in anti-PD-1/PD-L1 treated NSCLC patients, RASi use improved PFS and demonstrated a non-significant improvement in OS, possibly due to the small sample size [102]. In two larger ICI cohort studies, ARB users had significantly improved OS vs non-users, a stronger effect than other RASi agents [103].

Tumour microenvironment-activated ARBs (TMA-ARBs) were shown to more effectively reduce collagen, α-SMA, and solid stress in breast cancer models, compared to free ARBs. TMA-ARBs enhanced anti-PD-1 and anti-CTLA-4 efficacy in three murine models, including one previously non-responsive to ICIs, where cure rates reached 50% with combination therapy [82]. These TMA-ARBs also increased immune cell infiltration, polarised macrophages towards the anti-tumour M1 phenotype and decreased immunosuppressive CAF signalling molecules (CXCL3 and FasL) [82]. Likewise, losartan increased M1-TAMs, reduced M2 TAMs and improved anti-PD-1 efficacy via stromal remodelling in 4T1-Luc models [81].

Taken together, these results indicate that modulation of AT1R signalling by ARBs provides a broad potential to enhance cancer therapeutics across modalities. By normalising the TMA, inhibiting pro-oncogenic signalling, reducing drug toxicity and synergistically enhanced efficacy, ARBs show promise as combinatorial partners with targeted, cytotoxic, or immune-based therapies.

### Clinical evaluation of RASi in cancer

Despite substantial preclinical evidence implicating AT1R signalling in cancer progression and varied (but often positive) outcomes from meta-analyses of clinical observational studies and retrospective cohort studies, trials specifically designed to evaluate ARBs in oncology remain limited. Many trials have instead focused on ARB-mediated mitigation of cardiotoxicity associated with therapies such as doxorubicin or trastuzumab (Table 3). Initially, candesartan was shown to reduce cardiotoxic events in breast cancer patients receiving anthracycline therapy, with or without trastuzumab [104]. A follow up study found this effect to be less pronounced at 2 years, although some measurements were modestly protected [105]. No significant protective effect was shown by another study investigating candesartan adjuvant therapy in HER2+ breast cancer patients receiving trastuzumab, although candesartan treatment was not started until 3 months after anthracycline treatment [106]. Interestingly, AT1R and HER2 expression in breast cancer appears to be mutually exclusive [10], implying that candesartan may not function well in these cancers. The PRADA II study investigating the protective effects of valsartan or sacubitril/valsartan in anthracycline-induced heart dysfunction has added further data suggesting no cardioprotective effect of RASi in breast cancer [107] (Table 3).

**Table 3:** Clinical trial data on ARB use and cancer. Three primary studies designed to evaluate ARB use in cancer therapy showed mildly positive results.

Study Name & Reference	Cancer Type	Phase & Design	ARB Intervention	Key Outcomes
Prevention of Cardiac Dysfunction During Adjuvant Breast Cancer Therapy (PRADA) NCT01434134	Breast (Early invasive scheduled for anthracyclines treatment)	Phase II	Candesartan 32 mg	Candesartan group shown significantly less decline in left ventricular ejection fraction [104]
Prevention of Cardiac Dysfunction During Breast Cancer Therapy (PRADAII) NCT03760588	Breast (Early invasive scheduled for anthracyclines treatment)	Phase II	Sacubitril/valsartan (97/103 mg)	Anthracycline-based treatment for early breast cancer is associated with a reduction in left ventricular ejection fraction that was not significantly attenuated by sacubitril-valsartan. [107]
Evaluating the Effect of Candesartan vs Placebo in Prevention of Trastuzumab-associated Cardiotoxicity NCT00459771	Breast (HER2 + )	Phase III, RCT	Candesartan 32 mg/day during and post-trastuzumab	No significant benefit from the use of candesartan [106]
A phase 1 study of combination therapy with gemcitabine and candesartan in patients with pancreatic cancer (GECA-1) UMIN000002152	Pancreatic (unresecatble or recurrent)	Phase I, single arm open label	Candesartan 4 mg, 8 mg, 16 mg or 32 mg	Dose-limiting toxicity seen in 32 mg patients, with one patient at 16 mg showing grade 4 neutropenia [120]
A phase 2 study of combination therapy with gemcitabine and candesartan in patients with advanced or recurrent pancreatic cancer (GECA-2) UMIN000005580	Pancreatic (Advanced)	Phase II, Dose-escalation	Candesartan 8 mg or 16 mg + Gemcitabine	Improved progression-free survival with 16 mg dose (4.6 vs. 3.5 months, 16 vs 8 mg, respectively), 2/35 patients discontinued candesartan due to hypotension, PR in 11.4%, SD in 51.4% [108]
Proton w/ FOLFIRINOX-Losartan for Pancreatic Cancer NCT01821729	Pancreatic (Locally Advanced unresectable)	Phase II, Single arm	Losartan + FOLFIRINOX + chemoradiotherapy (proton)	Downstaging of locally advanced disease. Achieved complete surgical resection of 61% in locally advanced cases [109]
Losartan and Nivolumab in Combination with FOLFIRINOX and SBRT in Localized Pancreatic Cancer NCT03563248	Pancreatic (Locally advanced, borderline resectable)	Phase II, RCT	Losartan + FOLFIRINOX + SBRT + Nivolumab	No observed effect of losartan or losartan + nivolumab on R0 resection rate, PFS, OS or partial clinical response [110]
Losartan and Hypofractionated Rx After Chemo for Tx of Borderline Resectable or Locally Advanced Unresectable Pancreatic Cancer (SHAPER) NCT04106856	Pancreatic (Borderline resectable, locally advanced unresectable)	Phase I, Single arm	Losartan + Hypofractionated radiation therapy	Est. study completion: 2026-08-08 Aims to assess safety of losartan in combination with Hypofractionated radiation therapy, as well as hypotensive adverse events and patient reported QoL
Imaging Perfusion Restrictions from Extracellular Solid Stress - An Open-label Losartan Study (ImPRESS) NCT03951142	Brain (Primary and metastatic)	Phase II, Open label	Losartan	Delayed, Est. study completion: 2024-12-31 Aims to assess the impact of losartan on cerebral blood flow and solid stress as well as immunotherapy and/or radiotherapy
Losartan + Sunitinib in Treatment of Osteosarcoma NCT03900793	Osteosarcoma (Relapsed, refractory)	Phase I/Ib, Open label dose escalation	Losartan <50 mg up to <150 mg + Sunitinib	Est. study completion: 2027-02 Aims to assess dose-limiting toxicities of combination therapy, maximally tolerated dose and recommended phase 2 dose Secondary outcomes of anti-tumour activity
Losartan, Pembrolizumab and Stereotactic Body Radiation Therapy for the Treatment of Patients with Locally Recurrent, Refractory or Oligometastatic Head and Neck Squamous Cell Carcinoma NCT06211335	Head and neck squamous cell carcinoma (Recurrent, refractory or oligometastatic)	Phase I/Ib Single arm open label	Losartan + Pembrolizumab + SBRT	Est. study completion: 2027-06 Aims to assess incidence of TRAE, with secondary outcomes of ORR, DOR, OS, and PFS
PHL Treatment in Pancreatic Cancer NCT05365893	Pancreatic (resectable, non-metastatic, post-NAT, pre-surgery)	Phase I, parallel assignment	Losartan 50 mg/day + Paricalcitol + Hydroxychloriquine	Estimated study completion: 2026-12-31 Aims to assess incidence of TRAE and compare biological effect of PHL treatment in PDAC

Various cardiovascular trials with ARBs with cancer incidence as a secondary outcome showed neutral or increased cancer risk.

In pancreatic cancer, where preclinical data is particularly strong, clinical studies are exploring ARB combination therapies (Table 3). Candesartan with gemcitabine in advanced PDAC demonstrated a dose-dependent improvement in PFS, although this fell short of the 5-month PFS target [108]. Phase 1 dose escalation to 32 mg candesartan, which had been tolerated in HER2+ breast cancer patients, was not tolerated in advanced PDAC patients, potentially reflecting the higher burden of disease-related comorbidities in this cohort. Consequently, ARB combination therapy may warrant further evaluation in earlier-stage or locally advanced PDAC patients, where treatment tolerance and efficacy could differ from those observed in the metastatic setting.

More encouraging results were observed with losartan plus FOLFIRINOX (folinic acid, fluorouracil, irinotecan and oxaliplatin) and proton radiation in locally advanced, unresectable PDAC patients, achieving an R0 resection rate of 61%, approaching the 65% rate reported in the less advanced, borderline resectable patients not treated with losartan [109]. However, a randomised controlled trial combining losartan, nivolumab (anti-PD-1), FOLFIRINOX and stereotactic body radiation therapy (SBRT) found no significant improvement in R0 resection rates, PFS, OS or pathological completed response [110]. This discrepancy could reflect differences in radiation modality or surgical assessment criteria. Ongoing studies are investigating losartan in combination with paricalcitol and hydroxychloroquine to modify the TME and improve resectability, as well as with hypofractionated radiation in borderline or locally advanced disease, with quality of life as a secondary endpoint (Table 3). Despite inconsistent results, ARBs, particularly losartan, appear safe and moderately effective in combination with standard PDAC treatment regimens.

Beyond PDAC, ARB combinations are under investigation in other cancer types. In osteosarcoma, a phase 1 study evaluating losartan plus sunitinib in relapsed or refractory patients is underway, assessing preliminary anti-tumour activity (Table 3). The ability of ARBs to modulate the TME and enhance ICI efficacy is also being clinically assessed. In glioblastoma, losartan is under evaluation for its effects on cerebral blood flow, solid stress, and synergy with immune and radiotherapies, with supporting preclinical data showing efficacy in murine models [111]. Similarly, a phase 1 trial is assessing losartan, pembrolizumab (anti-PD-1) and SBRT in advanced head and neck squamous cell carcinoma (HNSCC), with secondary endpoints focusing on anti-tumour efficacy (Table 3).

While clinical evaluations of ARBs remain limited compared to preclinical and observational studies, existing evidence supports their safety and potential use in combination therapies. Pancreatic cancer has been the primary focus, particularly with chemoradiation, though results remain variable and are often limited by small sample sizes or design constraints. Although consistent clinical benefit has yet to be demonstrated, the capacity of ARBs to modulate the TME, especially in enhancing immunotherapeutic response, remains a compelling rationale for continued investigation.

## Discussion and future directions

Compelling preclinical evidence implicates AT1R signalling in key oncogenic processes fuelling significant interest in repurposing ARBs as anti-cancer therapeutics. ARBs have been shown in various pre-clinical models to inhibit several hallmarks of cancer across a wide range of cancer types, including many with unmet clinical need, via the suppression of key oncogenic signalling cascades (Fig. 1). ARB treatment reduces tumour burden, metastasis, angiogenesis, and may even prevent tumour initiation. Mechanistically these outcomes reflect the promotion of less invasive phenotypes, reduced pro-inflammatory cytokine expression and suppression of key signalling pathways (Fig. 1). Despite this, the translation of these preclinical successes into robust clinical benefits for patients remains an inconsistent and often frustrating endeavour.

### Barriers to clinical success

Clinical trials and retrospective analyses have yielded mixed results, with some suggesting ARBs but not ACEis offer protective effects, particularly in pancreatic cancer where losartan improved tumour resectability. However, trials are often underpowered or confounded by pooled analyses of RASis as a single drug class. There is also the suggestion that the dosage required to induce anti-cancer effects in humans is higher than can be tolerated. Even with ongoing trials, more robust data on the direct anti-cancer effects of ARB therapeutics in large cohorts are required.

#### Tumour heterogeneity

Cancer is a highly heterogenous disease, with different subtypes exhibiting distinct molecular profiles which are often dependent on different signalling cascades. RAS signalling may be more relevant in certain cancer subtypes, for example HER2 and AT1R expression appears to be mutually exclusive in breast cancer patients [10], indicating a clear need for biomarker-driven patient stratification.

#### RAS signalling complexity

The RAS is a complex signalling pathway [1] (Fig. 1). Inhibition of one component, e.g. AT1R, could trigger compensatory activation of parallel pathways such as EGFR signalling. Additionally, AT1R can transactivate other GPCRs [1], many of which remain poorly characterised in oncology. Further, the interplay between the ACE/Ang II/AT1R axis and the counter-regulatory ACE2/AT2R/Ang 1–7/MasR axis likely influence the various outcomes seen in studies. Not only is there an interplay between these axes, but also between the local and systemic RAS, which may interact to introduce further variability in response. Illuminating the precise interaction of the RAS components in a cancer specific context may allow further refinement of treatment strategies.

#### Dosing, drug delivery, and combination therapies

Whilst many preclinical studies have attempted to simulate clinically relevant therapeutic doses, many studies utilised supra-therapeutic ARB doses not feasible in patients. Achieving effective drug concentrations in the TME remains a challenge, compounded by tumour and stromal heterogeneity. Novel delivery strategies, such as TMA-ARBs, have shown preclinical promise by enhancing efficacy and limiting systemic toxicity [82]. ARBs demonstrate synergy with multiple anti-cancer agents, yet several rational combinations, including cisplatin and ICIs, remain clinically untested despite preclinical success [82, 99, 101].

### Unlocking the potential of AT1R inhibition in cancer

There has been 30 years of advancement since the approval of the first ARB, losartan [112]. While the repurposing of approved ARBs offers reduced development costs, established safety profiles and well characterised pharmacokinetics, exploration of alternative therapeutic modalities could unlock the therapeutic potential of AT1R inhibition and yield better patient outcomes.

#### Targeted drug delivery systems

Nanoparticle and liposome-based delivery systems, validated in mRNA vaccine platforms [113], could localise ARB activity to the TME, minimising systemic side effects and improving efficacy. Preclinical studies using pH-sensitive polymers linked to ARBs demonstrated targeted release at tumour sites, enhancing tumour response and reducing immunosuppression [82]. Such approaches may also allow dose escalation beyond the current maximum tolerated dose.

#### Peptide therapeutics

The first Ang II antagonist, saralasin, showed limited success due to poor stability and bioavailability [114]. However, modern peptide engineering may enable stable AT1R-binding peptides that either block signalling or bias the receptor to transactivate beneficial signalling pathways, potentially resulting in better protection against chemotherapy-induced cardiotoxicity while inhibiting cancer-related pathways.

#### Gene Therapy/RNAi interference and targeting multiple RAS components

Approaches such as RNA interference or gene therapy could offer sustained AT1R inhibition. Modulating multiple arms of the RAS, for example combining AT1R inhibition with Ang 1–7 treatment, could yield more robust anti-cancer effects [115]. Whilst there are several approved gene therapies, delivering these agents to the tumour cells remains a challenge.

#### Antibody therapeutics

Many monoclonal antibody (mAb) therapeutics are currently approved or in development for the treatment of cancer [106, 116]. The use of mAbs offers high affinity and selectivity, potentially overcoming any issues with off-target effects and improving receptor blockade compared to small-molecule inhibitor ARBs. One recombinant anti-AT1R mAb has been shown to inhibit breast cancer cell growth, in vitro and In vivo, to a greater extent than losartan [117]. Considering this increased efficacy vs losartan, it is possible that humanised or fully human, affinity matured anti-AT1R mAbs could have significant benefit on patient outcomes. Moreover, the recent development of nanobodies targeting AT1R that stabilise an inactive state of this receptor [118], resulting in comparable anti-hypertensive activity to losartan [119], could potentially be utilised in a cancer therapy context. In addition, modern antibody engineering techniques could generate anti-AT1R therapeutics with the capability to exert Fc-mediated effector functions (antibody-dependent cell-mediated cytotoxicity (ADCC), antibody-dependent cellular phagocytosis (ADCP), or complement-dependent cytotoxicity (CDC)) and harness the immune system to induce cancer cell death.

#### Further investigation

Whilst extensive research has been conducted into RAS, the role of AT1R signalling remains to be fully understood. Further studies are required to clarify its context-specific functions and address key unanswered questions before it can be fully established as an anti-cancer therapy. It may also be of benefit to investigate the potential of ARBs/AT1R targeting agents in high-risk populations as a preventative therapy.

#### Comprehensive preclinical evaluations

Characterisation of AT1R and interacting component expression across a wider range of cancer types and subtypes, including primary, metastatic, and stromal compartments is needed. Systematic analysis of a range of AT1R targeting treatments, including mAb therapies, in multiple preclinical models, in combination with standard-of-care therapies across an array of cancers would bolster current understanding. Molecular analysis of the role of AT1R, including downstream signalling pathways, receptor transactivation, interaction with other cancer-related proteins and cytokines, and effects on cell metabolism need to be undertaken to clarify disease-specific mechanisms.

#### Well-designed clinical trials

Whilst we await the results of key clinical trials in this field of research, further prospective, randomised, controlled clinical trials are required. Such trials would specifically be designed to evaluate the efficacy of AT1R modulation in well-defined cancer subtypes. Appropriate stratification of large cohorts based on AT1R and other RAS component expression levels and activity would allow better understanding of the role of AT1R in cancer. Utilising exploratory biomarker analyses within clinical trials would also allow identification of predictive markers of AT1R modulation.

Another consideration within these trials is the specific targeting of the TME with AT1R modulating agents to determine whether off-target effects can be prevented whilst exerting a potent anti-cancer impact. Analyses of immune cell infiltration, angiogenesis, and stromal remodelling would be valuable secondary endpoints in such trials. Investigating the role of AT1R in cancer, the use of novel therapeutic strategies and targeted drug delivery systems is key to the development of improved therapies needed to enhance current treatment options and improve outcomes for many cancer patients.

## Conclusion

The RAS, and specifically AT1R, is increasingly recognised as a significant player in a range of cancer-related processes across a variety of cancer types, making it a viable target for therapeutic intervention. Despite this, the full therapeutic potential of targeting AT1R has yet to be realised, making further research and clinical investigation essential. Numerous recent studies have demonstrated the overexpression of AT1R in malignant tissues versus benign and normal tissues, and its overexpression is frequently correlated with aggressive tumour characteristics and poorer overall survival.

Targeting AT1R with ARBs has shown promising preclinical results, namely, inhibiting tumour growth, inducing apoptosis, and reducing angiogenesis in various cancer models. However, the successful translation of these results into humans remains a challenge with limited or inconclusive data being generated to date (Table 3).

The RAS is a complex signalling network with multiple interacting components and feedback mechanisms and the oncogenic role of AT1R varies by cancer type and stage, making it difficult to predict how AT1R modulation will affect different patients. Therefore, future research must focus on clarifying the context-dependent role of AT1R in cancer as well as identify patient populations most likely to benefit from AT1R-targeted therapy. Well-designed clinical trials are essential to validate both the preclinical findings and the therapeutic value of ARBs in oncology. Development of new agents that more effectively modulate AT1R, such as mAbs, as well as utilisation of targeted drug delivery systems, may provide a more tenable option to reduce off target effects and improve patient outcomes.

## References

[1] Forrester SJ, Booz GW, Sigmund CD, Coffman TM, Kawai T, Rizzo V, et al. Angiotensin II Signal Transduction: An Update on Mechanisms of Physiology and Pathophysiology. Physiol Rev. 2018;98:1627–738. 29873596 10.1152/physrev.00038.2017 PMC6335102

[2] Pardhi TR, Karnik SS. Receptors | Angiotensin Receptors. In: JezJ, editor. Encyclopedia of Biological Chemistry III (Third Edition). Third Edition. Oxford: Elsevier; 2021. p. 110–21. Available from: https://www.sciencedirect.com/science/article/pii/B9780128194607000967.

[3] Shu Z, Wan J, Read RJ, Carrell RW, Zhou A Angiotensinogen and the Modulation of Blood Pressure. Front Cardiovasc Med. 2021 Mar 18 [cited 2025 Jan 13];8:645123. Available from: https://pmc.ncbi.nlm.nih.gov/articles/PMC8012498/. 10.3389/fcvm.2021.645123 PMC8012498 33816576

[4] Gironacci MM, Bruna-Haupt E. Unraveling the crosstalk between renin-angiotensin system receptors. Acta Physiologica. 2024;240:e14134. 38488216 10.1111/apha.14134

[5] Kirça M, Yeşilkaya A. Losartan inhibits EGFR transactivation in vascular smooth muscle cells. Turk J Med Sci. 2018;48:1364–71. 30543092 10.3906/sag-1802-113

[6] Gekle M, Eckenstaler R, Braun H, Olgac A, Robaa D, Mildenberger S, et al. Direct GPCR-EGFR interaction enables synergistic membrane-to-nucleus information transfer. Cell Mol Life Sci. 2024;81:272. 38900158 10.1007/s00018-024-05281-5 PMC11335197

[7] Hassani B, Attar Z, Firouzabadi N. The renin-angiotensin-aldosterone system (RAAS) signaling pathways and cancer: foes versus allies. Cancer Cell Int. 2023;23. 254. 37891636 10.1186/s12935-023-03080-9 PMC10604988

[8] Hanahan D, Weinberg RA The Hallmarks of Cancer. Cell. 2000;100:57–70. Available from: https://www.sciencedirect.com/science/article/pii/S0092867400816839. 10.1016/s0092-8674(00)81683-9 10647931

[9] Bray F, Laversanne M, Sung H, Ferlay J, Siegel RL, Soerjomataram I, et al. Global cancer statistics 2022: GLOBOCAN estimates of incidence and mortality worldwide for 36 cancers in 185 countries. CA Cancer J Clin. 2024 May [cited 2025 Jul 14];74(3):229–63. Available from: https://pubmed.ncbi.nlm.nih.gov/38572751/10.3322/caac.21834 38572751

[10] Ekambaram P, Lee JY, Hubel NE, Hu D, Yerneni S, Campbell PG, et al. The CARMA3-Bcl10-MALT1 signalosome drives NFκB activation and promotes aggressiveness in angiotensin II Receptor-Positive Breast Cancer. Cancer Res. 2018;78:1225–40. 29259013 10.1158/0008-5472.CAN-17-1089 PMC6436094

[11] Lozinski M, Lumbers ER, Bowden NA, Martin JH, Fay MF, Pringle KG, et al. Upregulation of the Renin–Angiotensin System Is Associated with Patient Survival and the Tumour Microenvironment in Glioblastoma. Cells. 2024;13:634. 38607073 10.3390/cells13070634 PMC11012120

[12] Lv J, Li L, Duan B. Hub Genes and Key Pathway Identification in Colorectal Cancer Based on Bioinformatic Analysis. Biomed Res Int. 2019 [cited 2025 Mar 29];2019. Available from: https://pubmed.ncbi.nlm.nih.gov/31781593/. 10.1155/2019/1545680 PMC6874977 31781593

[13] Ji Y, Chen H, Gow W, Ma L, Jin Y, Hui B, et al. Potential biomarkers Ang II/AT1R and S1P/S1PR1 predict the prognosis of hepatocellular carcinoma. Oncol Lett. 2020 Nov 1;20(5). 10.3892/ol.2020.12071 PMC7491028 32963614

[14] Li SH, Lu HI, Chang AYW, Huang WT, Lin WC, Lee CC, et al. Angiotensin II type I receptor (AT1R) is an independent prognosticator of esophageal squamous cell carcinoma and promotes cells proliferation via mTOR activation. Vol. 7. Available from: www.impactjournals.com/oncotarget. 10.18632/oncotarget.11567 PMC5341864 27564102

[15] Coulson R, Liew SH, Connelly AA, Yee NS, Deb S, Kumar B, et al. The angiotensin receptor blocker, Losartan, inhibits mammary tumor development and progression to invasive carcinoma. Vol. 8, Oncotarget. 2017. Available from: www.impactjournals.com/oncotarget/. 10.18632/oncotarget.15553 PMC5386636 28416734

[16] Li SH, Lu HI, Chang AYW, Huang WT, Lin WC, Lee CC, et al. Angiotensin II type I receptor (AT1R) is an independent prognosticator of esophageal squamous cell carcinoma and promotes cells proliferation via mTOR activation. Oncotarget. 2016;7:67150. 27564102 10.18632/oncotarget.11567 PMC5341864

[17] Arrieta O, Villarreal-Garza C, Vizcaíno G, Pineda B, Hernández-Pedro N, Guevara-Salazar P, et al. Association between AT1 and AT2 angiotensin II receptor expression with cell proliferation and angiogenesis in operable breast cancer. Tumor Biol. 2015;36:5627–34. 10.1007/s13277-015-3235-3 25682288

[18] Chen R, Hong Q, Jiang J, Chen X, Jiang Z, Wang J, et al. AGTR1 promoter hypermethylation in lung squamous cell carcinoma but not in lung adenocarcinoma. Oncol Lett. 2017;14:4989–94. 29085512 10.3892/ol.2017.6824 PMC5649578

[19] Yang Y, Zhang F, Skrip L, Lei H, Luo S, Lu K, et al. Lack of an association between angiotensin receptor blocker based therapy and increased risk of cancer: Evidence from large observational studies. PLoS One. 2015 Mar 19;10. 10.1371/journal.pone.0119775 PMC4366349 25790107

[20] Wang N, Liu J, Wang W, Qin J, Lin D The impact of Renin-angiotensin system blockers on lung cancers prognosis: A prisma-compliant systematic review and meta-analysis. 2017. Available from: http://www.alliedacademies.org/allied-journal-of-medical-research/.

[21] Zhu L, Li J, Qu X, Pang Z, Cui L, Shen H, et al. The impact of hypertension and renin-angiotensin system blockers on outcomes of lung cancer patients: a population-based retrospective cohort study. Vol. 10, Int J Clin Exp Pathol. 2017. Available from: www.ijcep.com/.

[22] Wei J, Zhou Z, Xu Z, Zeng S, Chen X, Wang X, et al. Retrospective clinical study of renin-angiotensin system blockers in lung cancer patients with hypertension. PeerJ. 2019;7:e8188. Available from: https://pubmed.ncbi.nlm.nih.gov/31844581/. 10.7717/peerj.8188 PMC6910116 31844581

[23] Sever N, Yunusov E, Çelebi A, Yaşar A, Majidova N, Kocaaslan E, et al. Impact of renin angiotensin system inhibitors on survival of patients with metastatic non-small cell lung cancer. Ann Saudi Med. 2025;45:18–24. 39929787 10.5144/0256-4947.2025.18 PMC11810880

[24] Chen X, Yi CH, Ya KG. Renin–angiotensin system inhibitor use and colorectal cancer risk and mortality: A dose–response meta analysis. JRAAS - Journal of the Renin-Angiotensin-Aldosterone System. 2020 Jul 1;21. 10.1177/1470320319895646 PMC7338647 32627649

[25] McKay RR, Rodriguez GE, Lin X, Kaymakcalan MD, Hamnvik OPR, Sabbisetti VS, et al. Angiotensin system inhibitors and survival outcomes in patients with metastatic renal Cell Carcinoma. Clin Cancer Res. 2015;21:2471–9. 25724518 10.1158/1078-0432.CCR-14-2332 PMC4566854

[26] Pinter M, Weinmann A, Wörns MA, Hucke F, Bota S, Marquardt JU, et al. Use of inhibitors of the renin–angiotensin system is associated with longer survival in patients with hepatocellular carcinoma. United European. Gastroenterol J. 2017;5:987–96. 10.1177/2050640617695698 PMC5676550 29163965

[27] Feng LH, Sun HC, Zhu XD, Zhang SZ, Li KS, Li XL, et al. Renin-angiotensin inhibitors were associated with improving outcomes of hepatocellular carcinoma with primary hypertension after hepatectomy. Ann Transl Med. 2019;7:739–739. 32042755 10.21037/atm.2019.11.131 PMC6990038

[28] Cheung KS, Chan EW, Seto WK, Wong ICK, Leung WKACE. Angiotensin-Converting Enzyme Inhibitors/Angiotensin Receptor Blockers Are Associated with Lower Colorectal Cancer Risk: A Territory-Wide Study with Propensity Score Analysis. Hypertension. 2020;76:968–75. 32623923 10.1161/HYPERTENSIONAHA.120.15317

[29] Wu CN, Wu SC, Chen WC, Yang YH, Chin JC, Chien CY, et al. Angiotensin II receptor blockers and oral squamous cell carcinoma survival: A propensity-score-matched cohort study. PLoS One. 2021 Dec 1;16. 10.1371/journal.pone.0260772 PMC8638984 34855858

[30] Izzedine H, Derosa L, Le Teuff G, Albiges L, Izzedine H. Hypertension and Angiotensin System Inhibitors: impact on outcome in Sunitinib treated patients for metastatic renal cell carcinoma. Annals of Oncology Advance Access. 2015. Available from: http://annonc.oxfordjournals.org/. 10.1093/annonc/mdv147 25795198

[31] Asgharzadeh F, Hashemzehi M, Moradi-Marjaneh R, Hassanian SM, Ferns GA, Khazaei M, et al. Angiotensin-converting enzyme inhibitors and angiotensin receptor blockers as therapeutic options in the treatment of renal cancer: A meta-analysis. Life Sci. 2020 Feb 1;242. 10.1016/j.lfs.2019.117181 31863771

[32] Keith SW, Maio V, Arafat HA, Alcusky M, Karagiannis T, Rabinowitz C, et al. Angiotensin blockade therapy and survival in pancreatic cancer: a population study. BMC Cancer. 2022 Dec 1;22. 10.1186/s12885-022-09200-4 PMC8819908 35130875

[33] Zhang J, Liu J, Chen J, Li X, Wu Y, Chen H, et al. Angiotensin receptor blockers (ARBs) reduce the risk of lung cancer: a systematic review and meta-analysis. Vol. 8, Int J Clin Exp Med. 2015. Available from: www.ijcem.com/. PMC4612864 26550179

[34] Busby J, McMenamin, Spence A, Johnston BT, Hughes C, Cardwell CR. Angiotensin receptor blocker use and gastro-oesophageal cancer survival: a population-based cohort study. Aliment Pharm Ther. 2018;47:279–88. 10.1111/apt.14388 29105106

[35] Facciorusso A, Del Prete V, Crucinio N, Muscatiello N, Carr BI, Di Leo A, et al. Angiotensin receptor blockers improve survival outcomes after radiofrequency ablation in hepatocarcinoma patients. J Gastroenterol Hepatol (Aust). 2015;30:1643–50. 10.1111/jgh.12988 25974743

[36] Smith L, Parris C, Veronese N, Shang C, López-Sánchez GF, Jacob L, et al. Cross-sectional associations between angiotensin-converting enzyme inhibitor use and cancer diagnosis in US adults. Clin Exp Med. 2020;20:409–16. 32219665 10.1007/s10238-020-00622-7

[37] Eskelinen T, Veitonmäki T, Kotsar A, Tammela TLJ, Pöyhönen A, Murtola TJ. Improved renal cancer prognosis among users of drugs targeting renin-angiotensin system. Cancer Causes Control. 2022;33:313–20. 34921656 10.1007/s10552-021-01527-w PMC8776666

[38] Miyajima A, Yazawa S, Kosaka T, Tanaka N, Shirotake S, Mizuno R, et al. Prognostic Impact of Renin–Angiotensin System Blockade on Renal Cell Carcinoma After Surgery. Ann Surg Oncol. 2015;22:3751–9. 25691280 10.1245/s10434-015-4436-0

[39] Sorich MJ, Kichenadasse G, Rowland A, Woodman RJ, Mangoni AA. Angiotensin system inhibitors and survival in patients with metastatic renal cell carcinoma treated with VEGF-targeted therapy: A pooled secondary analysis of clinical trials. Int J Cancer. 2016;138:2293–9. 26685869 10.1002/ijc.29972

[40] Jain A, Shah H, Simonsick EM, Metter EJ, Mangold L, Humphreys E, et al. Angiotensin receptor autoantibodies as exposures that modify disease progression: Cross sectional, longitudinal and in vitro studies of prostate cancer. J Transl Autoimmun. 2019;1:2. 10.1016/j.jtauto.2019.100008 PMC6953913 31930191

[41] Ni H, Rui Q, Zhu X, Yu Z, Gao R, Liu H. Antihypertensive drug use and breast cancer risk: A metaanalysis of observational studies. Oncotarget. 2017;8:62545–60. 28977968 10.18632/oncotarget.19117 PMC5617528

[42] Fatima K, Ellahi A, Adil M, Kashif H, Uzair M, Ashraf N, et al. The potential impact of renin-angiotensin system inhibitors on cancer survival and recurrence: A systemic review and meta-analysis. J Cardiovasc Pharmacol. 2024 [cited 2025 Jul 24]; Available from: https://journals.lww.com/cardiovascularpharm/fulltext/2025/01000/the_potential_impact_of_renin_angiotensin_system.4.aspx. 10.1097/FJC.0000000000001600 39027981

[43] Zhou T, Xie Y, Hou X, Bai W, Li X, Liu Z, et al. Irbesartan overcomes gemcitabine resistance in pancreatic cancer by suppressing stemness and iron metabolism via inhibition of the Hippo/YAP1/c-Jun axis. J Exp Clin Cancer Res. 2023;1:42. 10.1186/s13046-023-02671-8 PMC10157938 37143164

[44] Pei N, Mao Y, Wan P, Chen X, Li A, Chen H, et al. Angiotensin II type 2 receptor promotes apoptosis and inhibits angiogenesis in bladder cancer. J Exp Clin Cancer Res. 2017;9:36. 10.1186/s13046-017-0542-0 PMC5466725 28599664

[45] Liu Y, Li B, Wang X, Li G, Shang R, Yang J, et al. Angiotensin-(1–7) Suppresses Hepatocellular Carcinoma Growth and Angiogenesis via Complex Interactions of Angiotensin II Type 1 Receptor, Angiotensin II Type 2 Receptor and Mas Receptor. Mol Med. 2015;21:626. 26225830 10.2119/molmed.2015.00022 PMC4656199

[46] Hashemzehi M, Rahmani F, Khoshakhlagh M, Avan A, Asgharzadeh F, Barneh F, et al. Angiotensin receptor blocker Losartan inhibits tumor growth of colorectal cancer. EXCLI J. 2021;20:506–21. Available from: https://www.ncbi.nlm.nih.gov/pmc/articles/PMC8056058/. 10.17179/excli2020-3083 PMC8056058 33883980

[47] Zhang S, Wang Y. Telmisartan inhibits NSCLC A549 cell proliferation and migration by regulating the PI3K/AKT signaling pathway. Oncol Lett. 2018;15:5859–64. 29552215 10.3892/ol.2018.8002 PMC5840679

[48] Tsujiya Y, Hasegawa A, Yamamori M, Okamura N. Telmisartan-Induced Cytotoxicity via G2/M Phase Arrest in Renal Cell Carcinoma Cell Lines. Biol Pharm Bull. 2021;44:1878–85. 34853271 10.1248/bpb.b21-00654

[49] Zhang Q, Yu S, Lam MMT, Poon TCW, Sun L, Jiao Y, et al. Angiotensin II promotes ovarian cancer spheroid formation and metastasis by upregulation of lipid desaturation and suppression of endoplasmic reticulum stress. J Exp Clin Cancer Res. 2019;7:38. 10.1186/s13046-019-1127-x PMC6407256 30845964

[50] Ma Y, Xia Z, Ye C, Lu C, Zhou S, Pan J, et al. AGTR1 promotes lymph node metastasis in breast cancer by upregulating CXCR4/SDF-1α and inducing cell migration and invasion. Aging. 2019;19:11. 10.18632/aging.102032 PMC6628987 31219799

[51] Ji Y, Wang Z, Li Z, Zhang A, Jin Y, Chen H, et al. Angiotensin II Enhances Proliferation and Inflammation through AT1/PKC/NF-κB Signaling Pathway in Hepatocellular Carcinoma Cells. Cell Physiol Biochem. 2016;39:13–32. 27322819 10.1159/000445602

[52] Zhang GH, Miao FA, Xu JG, Zhang Y. Angiotensin II enhances the proliferation of Natural Killer/T-cell lymphoma cells via activating PI3K/Akt signaling pathway. Biosci Rep. 2020;13:40. 10.1042/BSR20202388 PMC7560539 32969473

[53] TSUJIYA Y, YAMAMORI M, HASEGAWA A, YAMAMOTO Y, YASHIRO M, OKAMURA N. Telmisartan exerts cytotoxicity in scirrhous gastric cancer cells by inducing g0/g1 cell cycle arrest. Anticancer Res. 2021;41:5461–8. 34732415 10.21873/anticanres.15358

[54] Guo R, Gu J, Zhang Z, Wang Y, Gu C. MicroRNA-410 functions as a tumor suppressor by targeting angiotensin II type 1 receptor in pancreatic cancer. IUBMB Life. 2015;67:42–53. 25646808 10.1002/iub.1342

[55] Li J, Wu X, Ni X, Li Y, Xu L, Hao X, et al. Angiotensin receptor blockers retard the progression and fibrosis via inhibiting the viability of AGTR1+ CAFs in intrahepatic cholangiocarcinoma. Clin Transl Med. 2023;13:e1213. Available from: https://www.ncbi.nlm.nih.gov/pmc/articles/PMC9975461/. ‌ 10.1002/ctm2.1213 PMC9975461 36855786

[56] Asgharzadeh F, Naghibzadeh N, Hashemzehi M, Mostafapour A, Hassanian SM, Avan A, et al. Angiotensin II Receptor Antagonist, Valsartan, Has Beneficial Effect in Lung Metastasis of Colorectal Cancer Treated with Fluorouracil. J Gastrointest Cancer. 2023;54:126–34. 35083728 10.1007/s12029-021-00717-8

[57] Tabatabai E, Khazaei M, Asgharzadeh F, Nazari SE, Shakour N, Fiuji H, et al. Inhibition of angiotensin II type 1 receptor by candesartan reduces tumor growth and ameliorates fibrosis in colorectal cancer. EXCLI J. 2021;20:863–78. 34121975 10.17179/excli2021-3421 PMC8192880

[58] Oura K, Tadokoro T, Fujihara S, Morishita A, Chiyo T, Samukawa E, et al. Telmisartan inhibits hepatocellular carcinoma cell proliferation in vitro by inducing cell cycle arrest. Oncol Rep. 2017;38:2825–35. 29048654 10.3892/or.2017.5977 PMC5780034

[59] Takiguchi T, Takahashi-Yanaga F, Ishikane S, Tetsuo F, Hosoda H, Arioka M, et al. Angiotensin II promotes primary tumor growth and metastasis formation of murine TNBC 4T1 cells through the fibroblasts around cancer cells. Eur J Pharmacol. 2021;15:909. 10.1016/j.ejphar.2021.174415 34375673

[60] Matsui T, Chiyo T, Kobara H, Fujihara S, Fujita K, Namima D, et al. Telmisartan inhibits cell proliferation and tumor growth of esophageal squamous cell carcinoma by inducing S-phase arrest in vitro and in vivo. Int J Mol Sci. 2019;1:20. 10.3390/ijms20133197 PMC6651359 31261874

[61] Oh E, Kim JY, Cho Y, An H, Lee N, Jo H, et al. Overexpression of angiotensin II type 1 receptor in breast cancer cells induces epithelial-mesenchymal transition and promotes tumor growth and angiogenesis. Biochim Biophys Acta Mol Cell Res. 2016;1863:1071–81. 10.1016/j.bbamcr.2016.03.010 26975580

[62] Alaaeldin R, Ali FEM, Bekhit AA, Zhao QL, Fathy M. Inhibition of NF-kB/IL-6/JAK2/STAT3 Pathway and Epithelial-Mesenchymal Transition in Breast Cancer Cells by Azilsartan. Molecules. 2022;1:27. 10.3390/molecules27227825 PMC9693603 36431925

[63] Nguyen L, Ager EI, Neo J, Christophi C. Regulation of colorectal cancer cell epithelial to mesenchymal transition by the renin angiotensin system. J Gastroenterol Hepatol (Aust). 2016;31:1773–82. 10.1111/jgh.13307 26849969

[64] Asgharzadeh F, Mostafapour A, Ebrahimi S, Amerizadeh F, Sabbaghzadeh R, Hassanian SM, et al. Inhibition of angiotensin pathway via valsartan reduces tumor growth in models of colorectal cancer. Toxicol Appl Pharmacol. 2022; 1:440. 10.1016/j.taap.2022.115951 35235860

[65] Matysiak-Burzyńska ZE, Nowakowska M, Domińska K, Kowalska K, Płuciennik E, Piastowska-Ciesielska AW. Silencing of angiotensin receptor 1 interferes with angiotensin II oncogenic activity in endometrial cancer. J Cell Biochem. 2018;119:9110–21. 30105775 10.1002/jcb.27174

[66] Sukumaran S, Patel HJ, Patel BM. Evaluation of role of telmisartan in combination with 5-fluorouracil in gastric cancer cachexia. Life Sci. 2016;154:15–23. 27117583 10.1016/j.lfs.2016.04.029

[67] Surapaneni SK, Nottingham E, Mondal A, Patel N, Arthur P, Gebeyehu A, et al. Telmisartan facilitates the anticancer effects of carp-1 functional mimetic and sorafenib in rociletinib resistant non-small cell lung cancer. Anticancer Res. 2021;41:4215–28. 34475041 10.21873/anticanres.15226 PMC8691118

[68] Ishizuka ET, Kanda A, Kase S, Noda K, Ishida S. Involvement of the receptor-associated prorenin system in the pathogenesis of human conjunctival lymphoma. Invest Ophthalmol Vis Sci. 2015;56:74–80. 10.1167/iovs.14-15743 25503453

[69] Huang P, Liang G, Jie X, Donghui W Angiotensin II type 2 receptor-interacting protein 3a inhibits ovarian carcinoma metastasis via the extracellular HMGA2-mediated ERK/EMT pathway. Tumor Biol. 2017;1:39. 10.1177/1010428317713389 28651497

[70] Liu ZL, Chen HH, Zheng LL, Sun LP, Shi L. Angiogenic signaling pathways and anti-angiogenic therapy for cancer. Signal Transduct Target Ther. 2023;8:1–39. 37169756 10.1038/s41392-023-01460-1 PMC10175505

[71] The Cancer Genome Atlas Program (TCGA) - NCI. [cited 2025 Jul 14]. Available from: https://www.cancer.gov/ccg/research/genome-sequencing/tcga.

[72] Del Bufalo D, Trisciuoglio D, Milella M. Crosstalk Between VEGF and Bcl-2 in Tumor Progression and Angiogenesis. In: Madame Curie Bioscience Database. Austin (TX): Landes Bioscience; 2013 [cited 2025 Apr 16]. Available from: https://www.ncbi.nlm.nih.gov/books/NBK6393/.

[73] Takagi H, Kaji K, Nishimura N, Ishida K, Ogawa H, Takaya H, et al. The angiotensin ii receptor blocker losartan sensitizes human liver cancer cells to lenvatinib-mediated cytostatic and angiostatic effects. Cells. 2021;10:1–16. 10.3390/cells10030575 PMC8001516 33807929

[74] Fan F, Tian C, Tao L, Wu H, Liu Z, Shen C, et al. Candesartan attenuates angiogenesis in hepatocellular carcinoma via downregulating AT1R/VEGF pathway. Biomedicine Pharmacother. 2016;83:704–11. 10.1016/j.biopha.2016.07.039 27470571

[75] Geng YL, Ding YJ, Ni L, Xu KD, Le VM, Ji R, Feng Y, et al. The role of angiotensin-(1-7) on acquired platinum resistance-induced angiogenesis in non-small cell lung cancer in vitro and in vivo. Neoplasma. 2021;68:770–9. 34034496 10.4149/neo_2021_201213N1347

[76] Araújo WF, Naves MA, Ravanini JN, Schor N, Teixeira VPC. Renin-angiotensin system (RAS) blockade attenuates growth and metastatic potential of renal cell carcinoma in mice. Urologic Oncol: Semin Original Investig. 2015;33:389.e1–389.e7. 10.1016/j.urolonc.2014.11.022 25595575

[77] Fu Y, Saxu R, Ridwan KA, Yao J, Chen X, Xu X, et al. Losartan Alleviates the Side Effects and Maintains the Anticancer Activity of Axitinib. Molecules. 2022;1:27. 10.3390/molecules27092764 PMC9101101 35566115

[78] Anderson NM, Simon MC The tumor microenvironment. Current Biology. 2020 Aug 17 [cited 2025 Apr 18];30(16):R921–5. Available from: https://www.cell.com/action/showFullText?pii=S0960982220309337. 10.1016/j.cub.2020.06.081 PMC8194051 32810447

[79] Shrestha S, Noh JM, Kim SY, Ham HY, Kim YJ, Yun YJ, et al. Angiotensin converting enzyme inhibitors and angiotensin II receptor antagonist attenuate tumor growth via polarization of neutrophils toward an antitumor phenotype. Oncoimmunology. 2015;5:e1067744. Available from: https://pubmed.ncbi.nlm.nih.gov/26942086/. ‌ 10.1080/2162402X.2015.1067744 PMC4760329 26942086

[80] Zhao Y, Cao J, Melamed A, Worley M, Gockley A, Jones D, et al. Losartan treatment enhances chemotherapy efficacy and reduces ascites in ovarian cancer models by normalizing the tumor stroma. Proc Natl Acad Sci USA. 2019;116:2210–9. 30659155 10.1073/pnas.1818357116 PMC6369817

[81] Zhao Q, He X, Qin X, Liu Y, Jiang H, Wang J, et al. Enhanced Therapeutic Efficacy of Combining Losartan and Chemo-Immunotherapy for Triple Negative Breast Cancer. Front Immunol. 2022 Jun 23;13. 10.3389/fimmu.2022.938439 PMC9259940 35812418

[82] Chauhan VP, Chen IX, Tong R, Ng MR, Martin JD, Naxerova K, et al. Reprogramming the microenvironment with tumorselective angiotensin blockers enhances cancer immunotherapy. Proc Natl Acad Sci USA. 2019;166:10674–80. 10.1073/pnas.1819889116 PMC6561160 31040208

[83] Shen Y, Wang X, Lu J, Salfenmoser M, Wirsik NM, Schleussner N, et al. Reduction of Liver Metastasis Stiffness Improves Response to Bevacizumab in Metastatic Colorectal Cancer. Cancer Cell. 2020;37:800–8.e7. 32516590 10.1016/j.ccell.2020.05.005

[84] Gu L, Zhu Y, Lee M, Nguyen A, Ryujin NT, Huang J, et al. Angiotensin II receptor inhibition ameliorates liver fibrosis and enhances hepatocellular carcinoma infiltration by effector T cells. 2023. Available from: 10.1101/2023.03.05.531188. 10.1073/pnas.2300706120 PMC10175751 37126700

[85] Oliveira MMB, de Araújo AA, Ribeiro SB, de Sales Mota PCM, Marques VB, da Silva Martins Rebouças C, et al. Losartan improves intestinal mucositis induced by 5-fluorouracil in mice. Sci Rep. 2021;1:11. 10.1038/s41598-021-01969-x PMC8636633 34853351

[86] Regan DP, Coy JW, Chahal KK, Chow L, Kurihara JN, Guth AM, et al. The Angiotensin Receptor Blocker Losartan Suppresses Growth of Pulmonary Metastases via AT1R-Independent Inhibition of CCR2 Signaling and Monocyte Recruitment. J Immunol. 2019;202:3087–102. 30971441 10.4049/jimmunol.1800619 PMC6504574

[87] Ito M, Oliverio MI, Mannon PJ, Best CF, Maeda N, Smithies O, et al. Regulation of blood pressure by the type 1 A angiotensin II receptor gene. Proc Natl Acad Sci USA. 1995;92:3521. 7724593 10.1073/pnas.92.8.3521 PMC42199

[88] Khan SU, Fatima K, Aisha S, Malik F. Unveiling the mechanisms and challenges of cancer drug resistance. Cell Commun Signal. 2024;22:109. 38347575 10.1186/s12964-023-01302-1 PMC10860306

[89] Cancel M, Fromont G, Blonz C, Chevreau C, Rioux-Leclercq N, Laguerre B, et al. Everolimus or sunitinib as first-line treatment of metastatic papillary renal cell carcinoma: A retrospective study of the GETUG group (Groupe d’Etude des Tumeurs Uro-Génitales). Eur J Cancer. 2021;158:1–11. 34619467 10.1016/j.ejca.2021.08.046

[90] Pottier C, Fresnais M, Gilon M, Jérusalem G, Longuespée R, Sounni NE. Tyrosine Kinase Inhibitors in Cancer: Breakthrough and Challenges of Targeted Therapy. Cancers (Basel). 2020;12:731. 32244867 10.3390/cancers12030731 PMC7140093

[91] Aydiner A, Ciftci R, Sen F. Renin-Angiotensin System Blockers May Prolong Survival of Metastatic Non-Small Cell Lung Cancer Patients Receiving Erlotinib. Med (US). 2015;94:e887. 10.1097/MD.0000000000000887 PMC4616356 26039117

[92] Miao L, Chen W, Zhou L, Wan H, Gao B, Feng Y Impact of Angiotensin I-converting Enzyme Inhibitors and Angiotensin II Type-1 Receptor Blockers on Survival of Patients with NSCLC. Sci Rep. 2016;17:6. 10.1038/srep21359 PMC4756359 26883083

[93] Rudd SG. Targeting pan-essential pathways in cancer with cytotoxic chemotherapy: challenges and opportunities. Cancer Chemother Pharm. 2023;92:241. 10.1007/s00280-023-04562-3 PMC10435635 37452860

[94] Cheng D, Tu W, Chen L, Wang H, Wang Q, Liu H, et al. MSCs enhances the protective effects of valsartan on attenuating the doxorubicin-induced myocardial injury via AngII/NOX/ROS/MAPK signaling pathway. Aging. 2021 Sep 29 [cited 2025 Apr 8];13(18):22556–70. Available from: https://www.aging-us.com/article/203569. 10.18632/aging.203569 PMC8507274 34587120

[95] Akolkar G, Bhullar N, Bews H, Shaikh B, Premecz S, Bordun KA, et al. The role of renin angiotensin system antagonists in the prevention of doxorubicin and trastuzumab induced cardiotoxicity. Cardiovasc Ultrasound. 2015 Apr 3;13. 10.1186/s12947-015-0011-x PMC4393607 25889218

[96] El-Said NT, Mohamed EA, Taha RA. Irbesartan suppresses cardiac toxicity induced by doxorubicin via regulating the p38-MAPK/NF-κB and TGF-β1 pathways. Naunyn Schmiedebergs Arch Pharm. 2019;392:647–58. 10.1007/s00210-019-01624-3 30734091

[97] Ghasemi M, Okay M, Turk S, Naeemaee R, Guver E, Malkan UY, et al. The impact of At1r inhibition via losartan on the anti-leukaemic effects of doxorubicin in acute myeloid leukaemia. JRAAS. 2019;20:1470320319851310. Available from: https://pubmed.ncbi.nlm.nih.gov/31117912/. ‌ 10.1177/1470320319851310 PMC6537254 31117912

[98] Matsushima-Otsuka S, Fujiwara-Tani R, Sasaki T, Ohmori H, Nakashima C, Kishi S, et al. Significance of intranuclear angiotensin-II type 2 receptor in oral squamous cell carcinoma. Oncotarget. 2018;9:36561–74. 30564297 10.18632/oncotarget.26337 PMC6290968

[99] Zhao B, Sun H, Zhang XD, Li GH, Zhao YH, Wang B. Potential anti-tumor mechanisms of renin angiotensin system inhibitors through inhibiting angiogenesis and influencing angiotensin II actions. Vol. 11, Int J Clin Exp Med. 2018. Available from: www.ijcem.com/.

[100] Menter AR, Carroll NM, Sakoda LC, et al. Effect of Angiotensin System Inhibitors on Survival in Patients Receiving Chemotherapy for Advanced Non–Small-Cell Lung Cancer. In: Clinical Lung Cancer. Elsevier Inc.; 2017. p. 189-197.e3. 10.1016/j.cllc.2016.07.008 PMC5424707 27637408

[101] Cheng Q, Zhou L, Zhou J, Wan H, Li Q, Feng Y. ACE2 overexpression inhibits acquired platinum resistance-induced tumor angiogenesis in NSCLC. Oncol Rep. 2016;36:1403–10. 27460845 10.3892/or.2016.4967

[102] Tozuka T, Yanagitani N, Yoshida H, Manabe R, Ogusu S, Tsugitomi R, et al. Impact of Renin–angiotensin System Inhibitors on the Efficacy of Anti-PD-1/PD-L1 Antibodies in NSCLC Patients. Anticancer Res. 2021;41:2093–100. 33813419 10.21873/anticanres.14980

[103] Mei J, Chu J, Yang K, Luo Z, Yang J, Xu J, et al. Angiotensin receptor blocker attacks armored and cold tumors and boosts immune checkpoint blockade. J Immunother Cancer. 2024;6:12. 10.1136/jitc-2024-009327 PMC11418576 39244215

[104] Gulati G, Heck SL, Ree AH, Hoffmann P, Schulz-Menger J, Fagerland MW, et al. Prevention of cardiac dysfunction during adjuvant breast cancer therapy (PRADA): a 2 × 2 factorial, randomized, placebo-controlled, double-blind clinical trial of candesartan and metoprolol. Eur Heart J. 2016;37:1671. 26903532 10.1093/eurheartj/ehw022 PMC4887703

[105] Heck SL, Mecinaj A, Ree AH, Hoffmann P, Schulz-Menger J, Fagerland MW, et al. Prevention of Cardiac Dysfunction during Adjuvant Breast Cancer Therapy (PRADA): Extended Follow-Up of a 2×2 Factorial, Randomized, Placebo-Controlled, Double-Blind Clinical Trial of Candesartan and Metoprolol. Circulation. 2021;143:2431–40. 33993702 10.1161/CIRCULATIONAHA.121.054698 PMC8212877

[106] Boekhout AH, Gietema JA, Kerklaan BM, VanWerkhoven ED, Altena R, Honkoop A, et al. Angiotensin II–Receptor Inhibition With Candesartan to Prevent Trastuzumab-Related Cardiotoxic Effects in Patients With Early Breast Cancer: A Randomized Clinical Trial. JAMA Oncol. 2016;2:1030–7. 27348762 10.1001/jamaoncol.2016.1726

[107] Omland T, Heck LS, Holte E, Lilleaasen MA, Gynnild NM, Fagerland WM. et al. Sacubitril/Valsartan and Prevention of Cardiac Dysfunction During Adjuvant Breast Cancer Therapy: The PRADA II Randomized Clinical Trial. Circulation. 2025;152:1136–1145. 40884047 10.1161/CIRCULATIONAHA.125.076616 PMC12537036

[108] Nakai Y, Isayama H, Ijichi H, Sasaki T, Takahara N, Ito Y, et al. A multicenter phase II trial of gemcitabine and candesartan combination therapy in patients with advanced pancreatic cancer: GECA2. Invest N Drugs. 2013;31:1294–9. 10.1007/s10637-013-9972-5 23690239

[109] Murphy JE, Wo JY, Ryan DP, Clark JW, Jiang W, Yeap BY, et al. Total Neoadjuvant Therapy with FOLFIRINOX in Combination with Losartan Followed by Chemoradiotherapy for Locally Advanced Pancreatic Cancer: A Phase 2 Clinical Trial. JAMA Oncol. 2019;5:1020–7. 31145418 10.1001/jamaoncol.2019.0892 PMC6547247

[110] Hong TS, Yeap BY, Horick NK, Wo JYL, Weekes CD, Allen JN, et al. A multicenter, randomized phase II study of total neoadjuvant therapy (TNT) with FOLFIRINOX (FFX) and SBRT, with or without losartan (L) and nivolumab (N) in borderline resectable (BR) and locally advanced (LA) pancreatic ductal adenocarcinoma (PDAC). J Clin Oncol. 2023;41:719–719.

[111] Datta M, Chatterjee S, Perez EM, Gritsch S, Roberge S, Duquette M, et al. Losartan controls immune checkpoint blocker-induced edema and improves survival in glioblastoma mouse models. Proc Natl Acad Sci USA. 2023;120:e2219199120. 36724255 10.1073/pnas.2219199120 PMC9963691

[112] Aulakh GK, Sodhi RK, Singh M. An update on non-peptide angiotensin receptor antagonists and related RAAS modulators. Life Sci. 2007;81:615–39. 17692338 10.1016/j.lfs.2007.06.007

[113] Tenchov R, Bird R, Curtze AE, Zhou Q. Lipid Nanoparticles from Liposomes to mRNA Vaccine Delivery, a Landscape of Research Diversity and Advancement. ACS Nano. 2021;15:16982–7015. 34181394 10.1021/acsnano.1c04996

[114] Adam M. Integrating research and development: the emergence of rational drug design in the pharmaceutical industry. Studies in History and Philosophy of Science Part C: Studies in History and Philosophy of Biological and Biomedical. Sciences. 2005;36:513–37. 10.1016/j.shpsc.2005.07.003 16137601

[115] Hinsley EE, de Oliveira CE, Hunt S, Coletta RD, Lambert DW. Angiotensin 1-7 inhibits angiotensin II-stimulated head and neck cancer progression. Eur J Oral Sci. 2017;125:247–57. 28653423 10.1111/eos.12356

[116] Al-Ali HN, Crichton SJ, Fabian C, Pepper C, Butcher DR, Dempsey FC, et al. A therapeutic antibody targeting annexin-A1 inhibits cancer cell growth in vitro and in vivo. Oncogene. 2024;43:608–14. 38200229 10.1038/s41388-023-02919-9 PMC10873194

[117] Redondo-Müller MA, Stevanovic-Walker M, Barker S, Puddefoot JR, Vinson GP. Anti-cancer actions of a recombinant antibody (R6313/G2) against the angiotensin II AT1 receptor. Endocr Relat Cancer. 2008;15:277–88. Available from: https://pubmed.ncbi.nlm.nih.gov/18310294/. 10.1677/ERC-07-0068 18310294

[118] Skiba MA, Sterling SM, Rawson S, Zhang S, Xu H, Jiang H, et al. Antibodies expand the scope of angiotensin receptor pharmacology. Nat Chem Biol. 2024;20:1577–85. 38744986 10.1038/s41589-024-01620-6 PMC11561159

[119] McMahon C, Staus DP, Wingler LM, Wang J, Skiba MA, Elgeti M, et al. Synthetic nanobodies as angiotensin receptor blockers. Proc Natl Acad Sci USA. 2020;117:20284–91. 32753386 10.1073/pnas.2009029117 PMC7443875

[120] Nakai Y, Isayama H, Ijichi H, Sasaki T, Kogure H, Yagioka H, et al. Phase I trial of gemcitabine and candesartan combination therapy in normotensive patients with advanced pancreatic cancer: GECA1. Cancer Sci. 2012;103:1489. 22515232 10.1111/j.1349-7006.2012.02311.x PMC7659287

